# Pan-Bcl-2 Inhibitor Obatoclax Delays Cell Cycle Progression and Blocks Migration of Colorectal Cancer Cells

**DOI:** 10.1371/journal.pone.0106571

**Published:** 2014-09-05

**Authors:** Bruno Christian Koehler, Anna-Lena Scherr, Stephan Lorenz, Christin Elssner, Nicole Kautz, Stefan Welte, Dirk Jaeger, Toni Urbanik, Henning Schulze-Bergkamen

**Affiliations:** National Center for Tumor Diseases, Department of Medical Oncology, Internal Medicine VI, Heidelberg University Hospital, Heidelberg, Germany; University of Illinois at Chicago, United States of America

## Abstract

Despite the fact that new treatment regimes have improved overall survival of patients challenged by colorectal cancer (CRC), prognosis in the metastatic situation is still restricted. The Bcl-2 family of proteins has been identified as promising anti cancer drug target. Even though small molecules targeting Bcl-2 proteins are in clinical trials, little is known regarding their effects on CRC. The aim of this study was to preclinically investigate the value of ABT-737 and Obatoclax as anticancer drugs for CRC treatment. The effects of the BH3-mimetics ABT-737 and Obatoclax on CRC cells were assessed using viability and apoptosis assays. Wound healing migration and boyden chamber invasion assays were applied. 3-dimensional cell cultures were used for long term assessment of invasion and proliferation. Clinically relevant concentrations of pan-Bcl-2 inhibitor Obatoclax did not induce cell death. In contrast, the BH3-mimetic ABT-737 induced apoptosis in a dose dependent manner. Obatoclax caused a cell line specific slowdown of CRC cell growth. Furthermore, Obatoclax, but not ABT-737, recovered E-Cadherin expression and led to impaired migration and invasion of CRC cells. The proliferative capacity and invasiveness of CRC cells was strikingly inhibited by low dose Obatoclax in long term 3-dimensional cell cultures. Obatoclax, but not ABT-737, caused a G1-phase arrest accompanied by a downregulation of Cyclin D1 and upregulation of p27 and p21. Overexpression of Mcl-1, Bcl-x_L_ or Bcl-2 reversed the inhibitory effect of Obatoclax on migration but failed to restore the proliferative capacity of Obatoclax-treated CRC cells. The data presented indicate broad and multifaceted antitumor effects of the pan-Bcl-2 inhibitor Obatoclax on CRC cells. In contrast to ABT-737, Obatoclax inhibited migration, invasion and proliferation in sublethal doses. In summary, this study recommends pan-Bcl-2 inhibition as a promising approach for clinical trials in CRC.

## Introduction

Colorectal carcinoma (CRC) represents the fourth common cause of death from cancer [1]. The incidence is increasing worldwide and 40% of all patients have distant organ metastasis at the time of first diagnosis. Systemic therapy approaches and surgery have improved overall survival, but the prognosis in UICC stage IV is still poor. The Bcl-2 protein family comprises key regulators of apoptosis acting at the mitochondrial surface. Antiapoptotic members of the family act by binding their proapoptotic relatives, thereby protecting the cell from death. The antiapoptotic proteins Mcl-1, Bcl-2 and Bcl-x_L_ have been shown to be upregulated in several solid and hematological cancer entities including CRC [2]–[4]. These observations led to the investigation of several compounds directly targeting antiapoptotic Bcl-2 proteins. So called BH3-mimetics act by binding to the BH3 cleft of antiapoptotic proteins [5]. This interaction leads to the release of proapoptotic Bcl-2 proteins finally promoting cell death. Clinical trials have proven the safety and efficacy of BH3 mimetics in various hematological and few solid malignancies [6]. Despite clinical investigations, valid preclinical data on the potency of BH3 mimetics as a treatment option for CRC are limited. In this study, we investigated the antitumoral activity of Obatoclax and ABT-737 on CRC cells. Both are small molecule inhibitors of antiapoptotic Bcl-2 proteins. They differ in their profile of inhibition, since ABT-737 does not inhibit Mcl-1 whereas Obatoclax is a pan-Bcl-2 inhibitor. Our study shows that ABT-737 induces cell death in various CRC cell lines. In contrast, the cell death inducing capacity of Obatoclax is limited and varies among CRC cell lines.

The capacity to migrate and to invade foreign tissues is a common feature of cancer cells dramatically contributing to the malignancy of the disease. Our group has recently demonstrated that downregulation of Mcl-1, Bcl-x_L_ or Bcl-2 leads to a striking impairment of migration and invasion of CRC cells [7]. Here, we investigate the relevance of the BH3-mimetics Obatoclax and ABT-737 for those malignant features. In contrast to ABT-737, sublethal doses of Obatoclax block migration and invasion of CRC cells in a Bcl-2 protein dependent manner.

Additionally, this study aims to assess the proliferative capacity of CRC cells treated with Obatoclax and ABT-737. Low dose Obatoclax, but not ABT-737, has impressive inhibitory effects on cell cycle progression and proliferation. Here, we describe antiproliferative effects of Obatoclax independent of Bcl-2 proteins.

In conclusion, our data revealed that pan-Bcl-2 inhibitor Obatoclax exerts various antitumor activities independent of cell death induction, recommending pan-Bcl-2 inhibition for further clinical development in colorectal cancer.

## Results

### ABT-737 but not Obatoclax mesylate induces apoptosis in CRC cells

**Figure 1:**
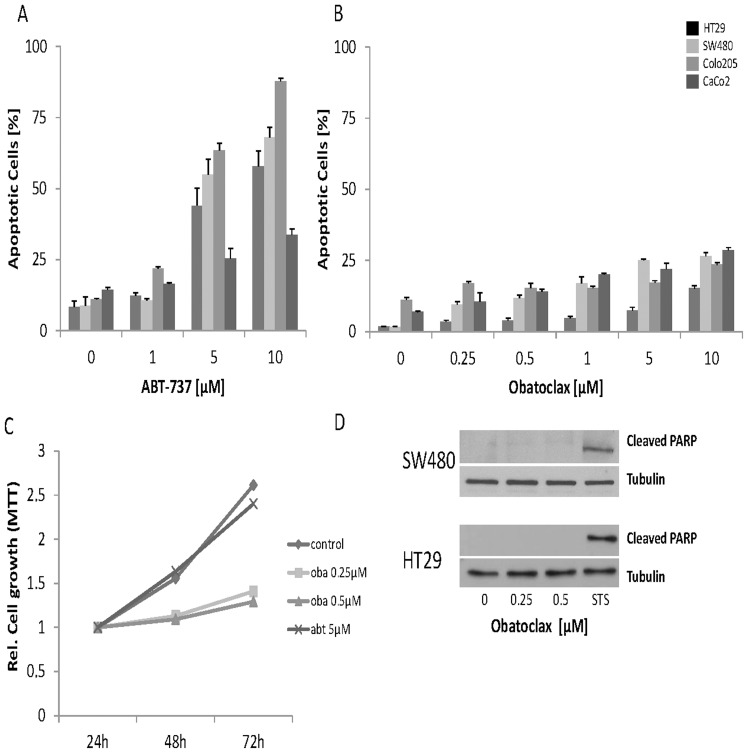
CRC cell growth, viability and death under BH3-mimetic treatment. (**A and B**) Flow cytometric analysis for DNA-fragmentation as an indicator for apoptotic death in four CRC cell lines; 48 h treatment with ABT-737 or Obatoclax. (**C**) MTT assay of HT29 cells after 72 h of Obatoclax and ABT-737 treatment. (P-values: Oba 0.25 µM: 0.003; Oba 0.05 µM: 0.004; ABT-737 5 µM: 0.589) (**D**) Representative Western blot for cleaved PARP after 24 h of Obatoclax treatment. Tubulin served as loading control. 2 µM treatment with Staurosporine for 24 h served as a positive control for cell death induction. Assays were performed in triplicates. Bars represent mean ± SD. Assays are representative of at least three independent experiments. Oba  =  Obatoclax, STS  =  Staurosporine.

To assess cell death after treatment of CRC cells with Obatoclax and ABT-737 for 48 h, we analyzed DNA fragmentation by flow cytometry. ABT-737 caused cell death in all cell lines investigated in a dose-dependent manner. The most striking effect was observed in Colo205 cells (more than 90% apoptotic cells after 48 h treatment with 10 µM ABT-737, Fig. 1A). In contrast, increasing concentrations of Obatoclax only slightly induced cell death in SW480, Colo205 and CaCo2 cells.

HT29 cells showed no apoptotic DNA fragmentation (Fig. 1B).

We further verified our findings by Western blotting for cleaved PARP. In line with flow cytometry data, there was no cleaved PARP detectable in all CRC cell lines as exemplarily shown for HT29 and SW480 in Fig. 1D.

In order to investigate effects of Obatoclax on proliferation, we followed cell growth of HT29 cells over time (72 h). Cell growth was inhibited even under low dose Obatoclax, whereas untreated and ABT-737 treated cells continued to proliferate. This result is indicative for a strong effect of Obatoclax on the proliferative capacity (Fig 1C).

Next, we aimed to assess the potency of Obatoclax alongside chemotherapeutic agents approved for CRC treatment. We observed that Oxaliplatin's cytotoxicity was increased when combined with Obatoclax. Effects are shown for a treatment period of 48 h in conventional cell culture and further validated for a treatment period of 7 days in 3D cell culture (Fig. S3). Neither Obatoclax nor Oxaliplatin alone were able to induce cell death in SW480 cells (Percentage of cleaved PARP positive cells: 1.3% [untreated], 1.7 [0.25 µM Obatoclax] and 1.6% [20 µM Oxaliplatin]). In striking contrast, 22.7% of cells underwent apoptosis as indicated by cleaved PARP staining when Obatoclax and Oxaliplatin were combined (Fig. S3C). Importantly, there was no sensitizing effect for the combination of Obatoclax with 5-FU (data not shown). Taken together, our observations indicate a dose dependent cell death induction by ABT-737 and a dose and cell type dependent effect on proliferation of Obatoclax. The combination of Bcl-2 inhibitors with chemotherapy, e.g. oxaliplatin, should be analysed as a potential treatment approach in future studies.

### Obatoclax recovers E-Cadherin in CRC cells, but leaves antiapoptotic Bcl-2 protein levels unchanged

**Figure 2:**
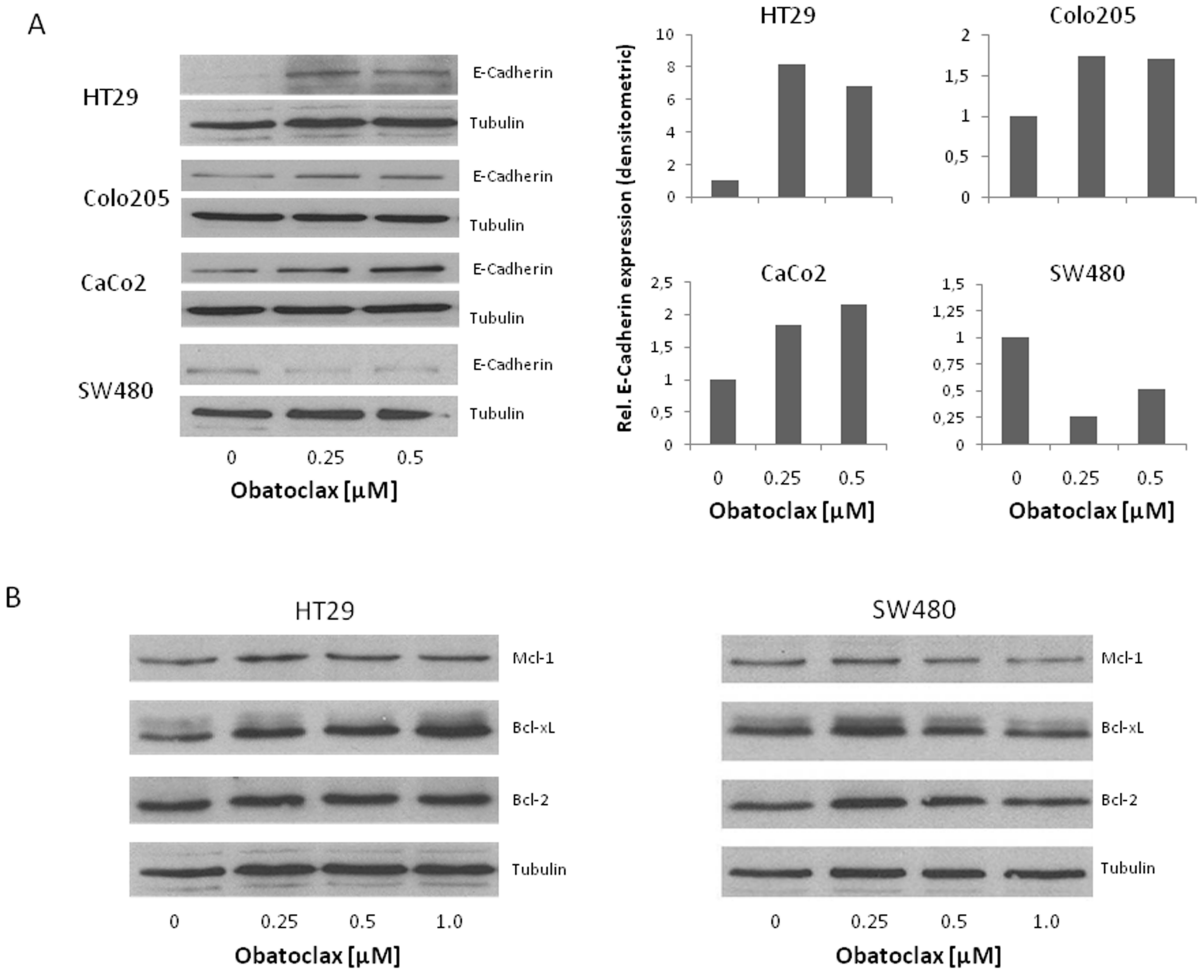
Western blot analysis for E-Cadherin and antiapoptotic Bcl-2 proteins in CRC cells treated with Obatoclax. (**A**) Western blotting for E-Cadherin in four CRC cell lines after 24 h treatment with Obatoclax in escalating doses (left). Corresponding densitometric analysis relative to untreated controls and adjusted to Tubulin as loading control. (**B**) Western blotting for Mcl-1, Bcl-2 and Bcl-x_L_ in HT29 cells (left) and SW480 cells (right) after 24 h treatment with Obatoclax. Tubulin served as a loading control. Western blots are representative for at least three blots from independent experiments.

We have recently demonstrated that siRNA-mediated downregulation of antiapoptotic Bcl-2 proteins impairs migration and invasion of CRC cells [7]. E-Cadherin is a primarily membrane bound protein with a key function for adherence junctions and Wnt-signaling. It has been shown to be lost during malignant transformation [8]. Impressively, we found a prominent recovery of E-Cadherin in all cell lines, except for SW480 cells, after Obatoclax treatment (Fig. 2A). Of note, N-Cadherin, which has been reported as a promoter of migration, was not detectable in the four cell lines investigated (data not shown) [9].

Others reported that antiapoptotic Bcl-2 proteins, e.g. Mcl-1, were rapidly and completely degraded in cancer cells treated with Obatoclax [10]. In sharp contrast, our data show no decrease of Mcl-1, Bcl-2 or Bcl-x_L_ levels. Quite contrary, whole cell protein immunoblotting revealed an increased level of Bcl-x_L_ and a slight increase of Bcl-2 levels in HT29 cells for all Obatoclax concentrations applied (Fig. 2B, left). In SW480 cells, Bcl-2 and Bcl-x_L_ levels increased under low dose Obatoclax, but showed levels similar to untreated cells under higher Obatoclax doses (Fig. 2B, right). Mcl-1 levels showed a more prominent increase under Obatoclax treatment compared to Bcl-2 and Bcl-x_L_ in HT29 cells (Fig. 2B, left). No remarkable changes in Mcl-1 levels were detected for SW480 cells (Fig. 2B, right).

### Low dose Obatoclax strikingly impairs migration and invasion of CRC cells

**Figure 3:**
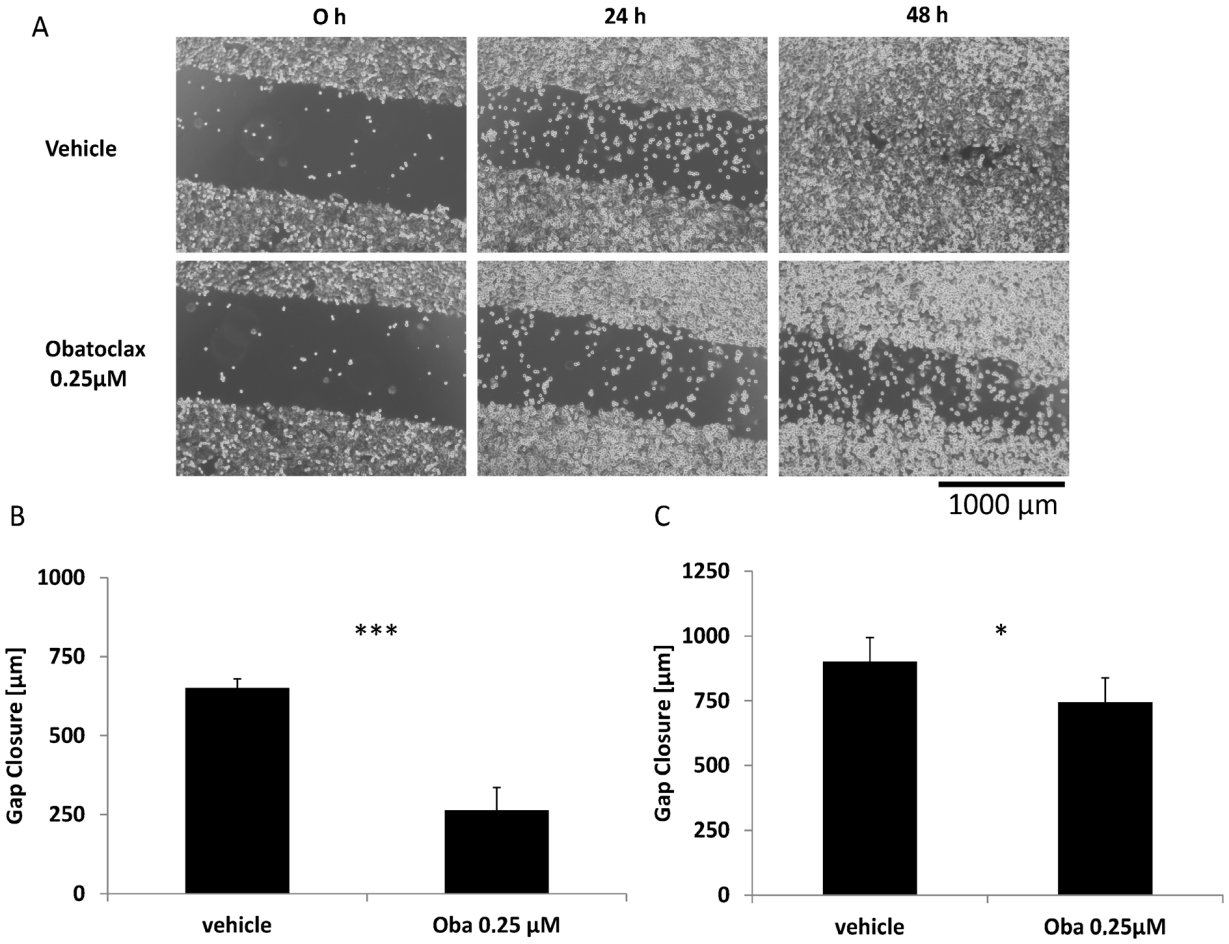
Migration of CRC cells treated with Obatoclax. (**A**) Representative pictures of wound healing scratch assays of vehicle (upper) and Obatoclax (lower) treated HT29 cells. Scale bar apply for all pictures. (**B**) Gap closure of HT29 cells after 48 h treatment with Obatoclax. (**C**) Gap closure of CaCo2 cells treated 48 h with Obatoclax. Assays were performed in triplicates. Bars represent mean ± SD. Assays are representative of at least three independent experiments. *p<0.05, ***p<0.001. Oba  =  Obatoclax.

In order to further investigate the impact of Obatoclax on CRC cells, we performed wound healing migration assays. Even sublethal doses were able to massively impair migratory capacity of HT29 cells over time. After 48 h, the measured gap closure was 650 µm in control cells compared to 263 µm in Obatoclax treated cells (Fig. 3A and B, p<0.001). In addition, CaCo2 cells migrated significantly less under treatment with 0.25 µM Obatoclax. Gap closure was 901 vs. 744 µm (Fig. 3C, p<0.05). Of note, ABT-737 failed to inhibit migration even in a dose of 5 µM as shown in Fig. S1.

**Figure 4:**
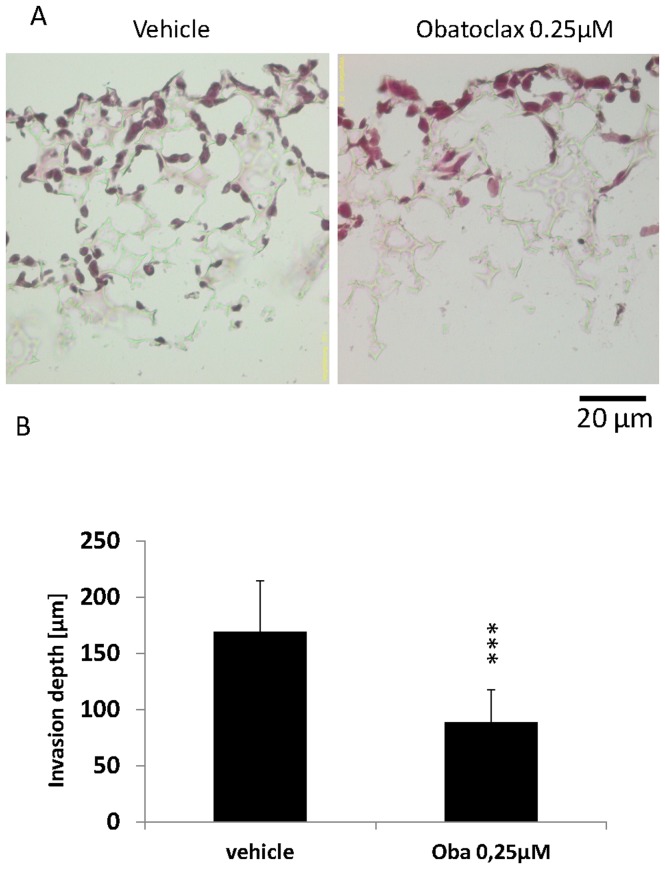
Migration of CRC cells treated with Obatoclax for 7 days in 3D scaffolds. (**A**) Representative pictures of HT29 cells in scaffolds after 7 days treatment with Obatoclax (Hematoxylin and Eosin staining. Scale bar applies for both pictures) (**B**) Corresponding analysis of invasion depth in scaffolds. Assays were performed in triplicates. Bars represent mean ± SD. Assays are representative of at least three independent experiments. ***p<0.001. Oba  =  Obatoclax.

Three-dimensional cell culture systems better reflect physiological cell growth as well as morphology and foster cell-cell interactions [11]. Furthermore, a three-dimensional system raises the possibility to perform long time cell culture experiments including drug exposure gaining more information than conventional cell culture. Thus, we used polystyrol scaffolds for 7 days of Obatoclax treatment followed by assessment of invasion, proliferation and apoptosis. HT29 cells did not show induction of apoptosis after treatment with Obatoclax (Fig. 1B and D). Strikingly, there was a massive blockade of invasion in 3D long term cell culture (Fig. 4A and B). In addition, Colo205 showed a profoundly impaired migration in long term cell culture as indicated by a decrease of invasion depth (Fig. S2). No apoptosis induction but an impairment of proliferation, as indicated by a reduction of Ki67 positivity, was observed (Fig. S2). This observation further underlines the broad antitumor effectiveness of Obatoclax with regard to a migration inhibitory phenotype combined with an antiproliferative effect, independent of cell death induction.

**Figure 5:**
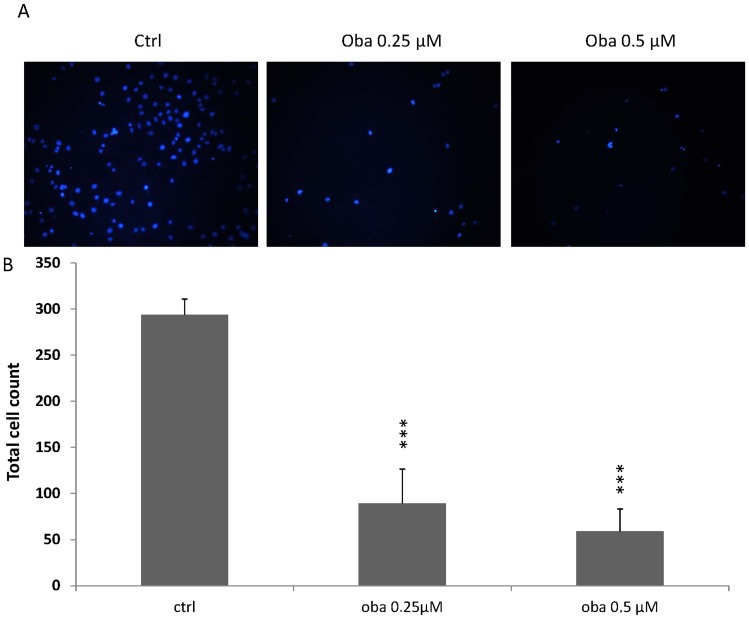
Invasion of SW480 cells treated with Obatoclax for 72 h in Matrigel coated Boyden chambers. SW480 cells were seeded into the upper chamber of a transwell. 48 h after seeding, nuclei on the lower surface were visualized by Hoechst staining. (**A**) Representative pictures of lower insert surface after Hoechst staining (scale bar indicate magnification for all panels). (**B**) Five fields of view per insert were counted. N = 5 per group. Values are expressed as mean ± SD. Assays are representative of at least three independent experiments. ***p<0.001. Oba  =  Obatoclax, ctrl  =  control.

Next, we investigated the invasiveness of SW480 cells treated with low dose Obatoclax in a matrigel containing boyden chamber assay. In order to prove appropriate attachment of cells in Obatoclax containing medium, cells were previously seeded onto polystyrol plates followed by MTT assay after 24 h. There was no impaired attachment observed in the presence of Obatoclax (data not shown). Invasion was strikingly inhibited in cells treated with 0.25 µM Obatoclax (293 invaded control cells vs. 89 invaded Obatoclax treated cells, p<0.001, Fig. 5) and was further abrogated in cells treated with 0.5 µM Obatoclax (293 invaded control cells vs. 59 invaded Obatoclax treated cells, p<0.001, Fig. 5).

### Overexpression of antiapoptotic Bcl-2 proteins restores migration of Obatoclax treated CRC cells

**Figure 6:**
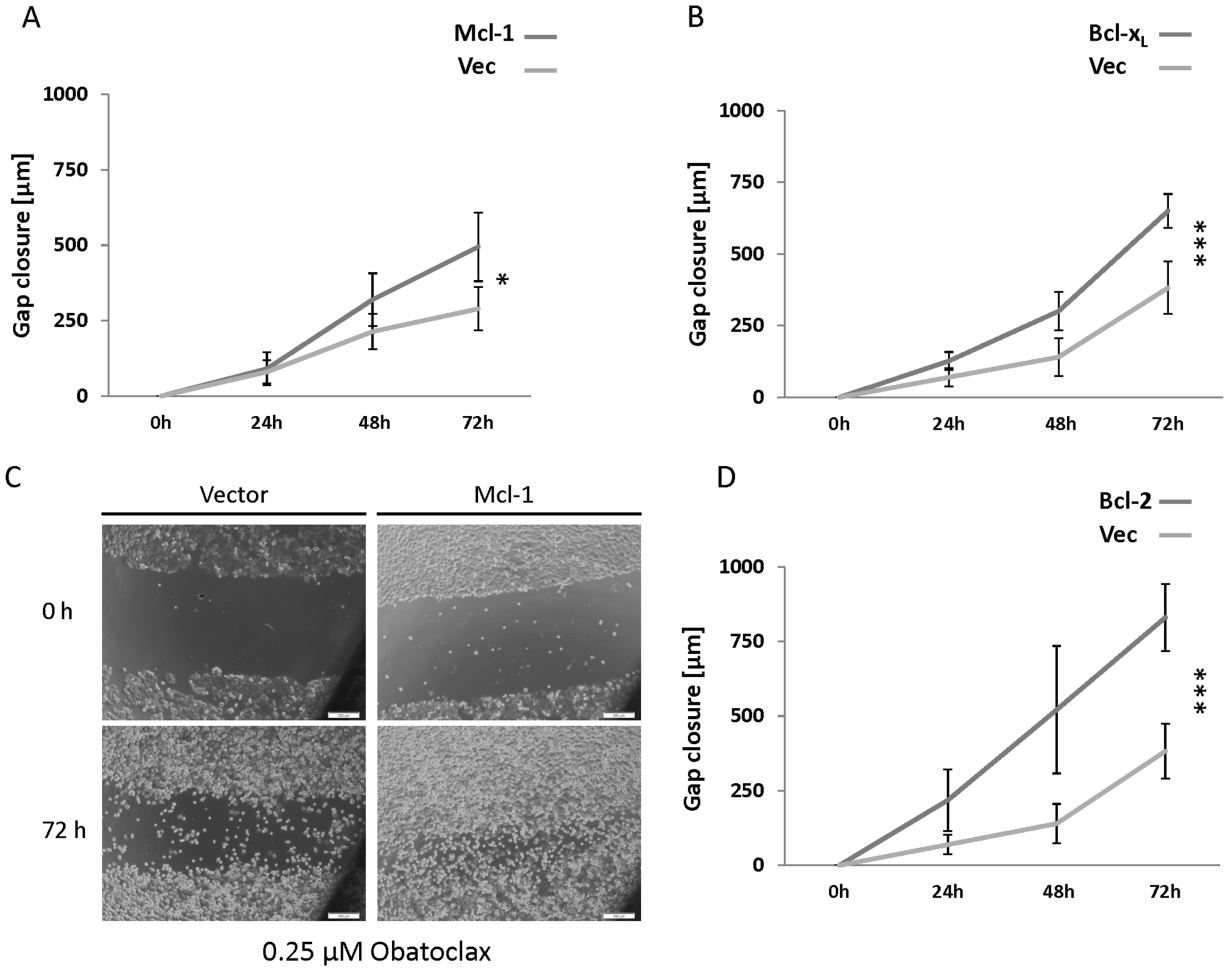
Migration of HT29 cells overexpressing antiapoptotic Bcl-2 proteins and treated with Obatoclax. (**A–D**) Gap closure of wound healing migration assays of HT29 cells treated with Obatoclax. (**A**) Vector and Mcl-1 transfected cells (**B**) vector and Bcl-xL transfected cells and (**D**) vector and Bcl-2 transfected cells. (**C**) Representative pictures of wound healing of vector and Mcl-1 transfected cells treated with Obatoclax. Values are expressed as mean ± SD. Assays are representative of at least three independent experiments. ***p<0.001. Vec  =  vector.

Next, we aimed to explore the commitment of antiapoptotic Bcl-2 proteins to the migration inhibitory phenotype caused by Obatoclax. We found an impressive recovery of migration in HT29 cells treated with 0.25 µM Obatoclax but overexpressing antiapoptotic Bcl-2 proteins. This phenotype reverting effect was observed for Mcl-1 (p<0.05), Bcl-x_L_ (p<0.001) and Bcl-2 (p<0.001), with the most pronounced effect for Bcl-2 (Fig. 6A–D). Overexpression of antiapoptotic proteins constantly blocked the inhibition of migration under Obatoclax treatment over 72 h. It is of great importance in this context to ensure that a restoration of migration is no secondary feature of an increased proliferation due to an overexpression of antiapoptotic Bcl-2 proteins. Therefore, we investigated proliferation in cells overexpressing Mcl-1, Bcl-2 or Bcl-x_L_. We observed no effect on proliferation for any of the proteins investigated as shown in detail in Fig. S4. (A–D).

### Low dose Obatoclax inhibits proliferation via G1-Phase arrest accompanied by an upregulation of p27 and p21 as well as downregulation of Cyclin D1

**Figure 7:**
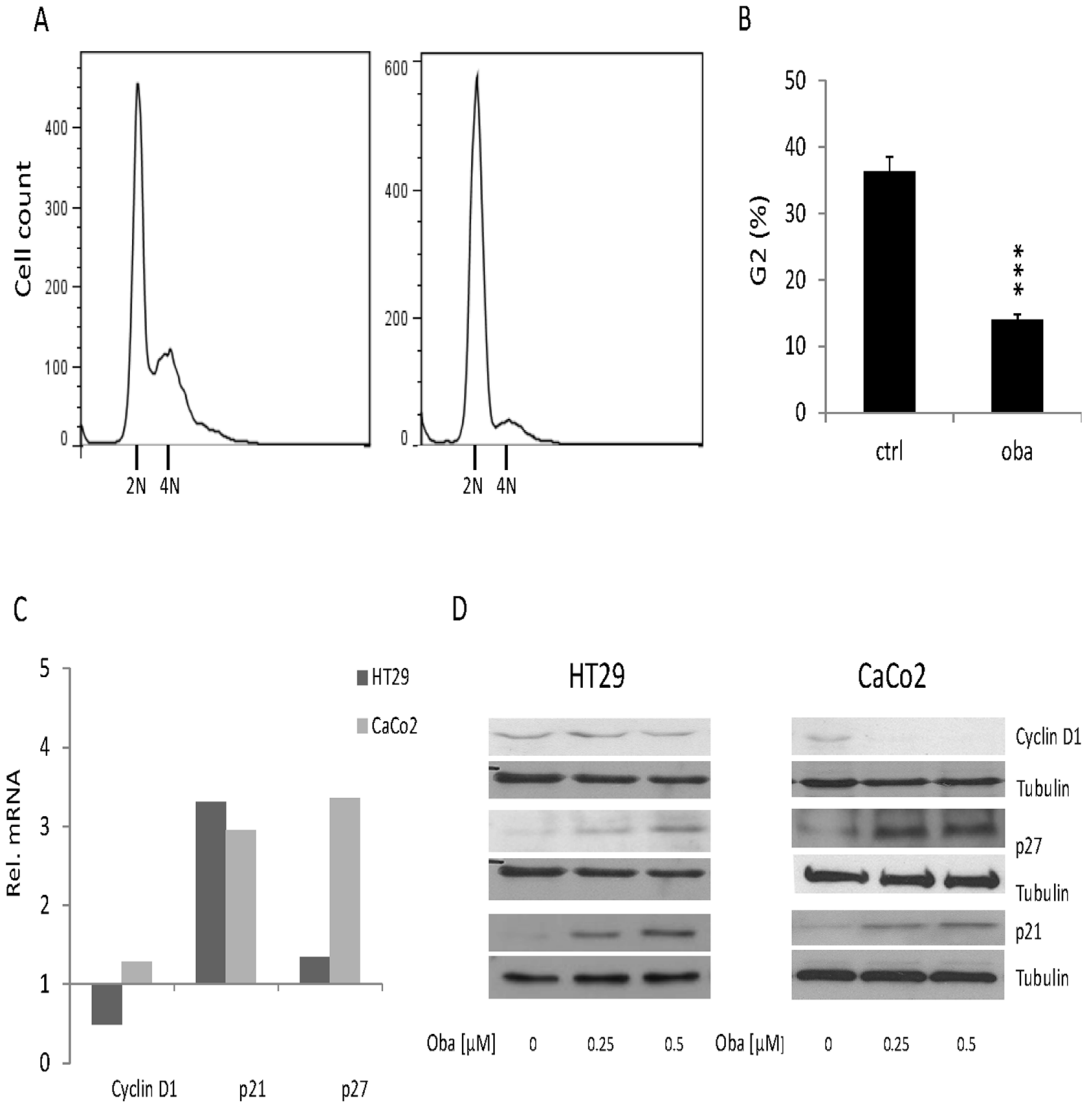
Cell cycle and proliferation analysis of CRC cells treated with Obatoclax. (**A**) Representative flow cytomeric analysis for DNA content in HT29 cells treated with 0.25 µM Obatoclax. 2N =  diploid cells in G1-phase, 4N =  tetraploid cells in G2-Phase. (**B**) Graphical analysis of cell cycle phase distribution corresponding to (**A**). Values are expressed as mean ± SD. ***p<0.001. (**C**) Rel. mRNA levels of Cyclin D1, p21 and p27 in HT29 and CaCo 2 cells after 24 h treatment with Obatoclax. mRNA levels were quantified by qrT-PCR and normalized to GAPDH as housekeeping gene. Assays are representative of at least three independent experiments. (**D**) Representative Western blots for Cyclin D1, p21, and p27 in HT29 and CaCo2 cells treated with Obatoclax for 24 h. Tubulin served as a loading control. Oba  =  Obatoclax, ctrl  =  control.

Since CRC cells in 3D scaffolds showed an impairment of proliferation under long term treatment with Obatoclax, we decided to further dissect cell cycle regulation. Staining for DNA content under Obatoclax treatment revealed a massive shift from the G2- in the G1-Phase of the cell cycle which is indicative for G1-phase arrest or a disrupted G1-phase transition. The proportion of cells in the G2-phase decreased from 37% in control cells to 13% in HT29 cells treated with 0.25 µM Obatoclax (Fig. 7A and B, p<0.001).

In addition, we aimed to quantify central cell cycle regulatory proteins under Obatoclax treatment. Cyclin D1 (CD1) represents the key Cyclin for G1-Phase transition [12]. CD1 was markedly downregulated under Obatoclax treatment on both the mRNA and protein level in HT29 (0.5 fold change) and CaCo2 cells (more than 0.5 fold change, Fig. 7C and D). p27 is a Cyclin dependent kinase inhibitor (CDKI) and its upregulation indicates cell cycle arrest [13]. We observed an upregulation of p27 on mRNA and protein levels in HT29 and CaCo2 cells treated with Obatoclax (Fig. 7C and D). In addition, we observed a remarkable and dose dependent increase of Cyclin dependent kinase inhibitor p21 on mRNA and protein levels in HT29 and CaCo2 cells (Fig. 7C and D). Taken together, these data are indicative for a cell cycle regulation by Obatoclax via key regulatory proteins of cell cycle transition, such as Cyclin D1, p27 and p21.

## Discussion

Anitapoptotic members of the Bcl-2 protein family are frequently overexpressed in human cancers including CRC [3]. High levels of antiapoptotic proteins have been shown to contribute to poor therapy response and to promote tumor progression. For instance, Bcl-x_L_ expression correlates with lymph node metastasis, poor differentiation and higher Duke's stage in CRC [14]. Expression patterns of Mcl-1 are predictive for therapy response in patients diagnosed with metastasized CRC [15]. Therefore, great efforts have been made in order to target antiapoptotic Bcl-2 proteins aiming at cancer cell death induction [16]. Importantly, deeper mechanistic and structural insights led to the development of small molecule inhibitors of antiapoptotic Bcl-2 proteins. This class of small molecules acts by binding to the hydrophobic BH3-cleft of antiapoptotic Bcl-2 proteins thereby mimicking proapoptotic Bcl-2 proteins and promoting cell death [17]. Safety and dose finding trials with Obatoclax have been carried out in solid malignancies and lymphoma [18], [19]. Despite the fact that BH3-mimetics have already entered early clinical trials, only few is known about the effects of Bcl-2 protein inhibition apart from cell death regulation [18], [19]. We have recently shown that a knockdown of Mcl-1, Bcl-2 or Bcl-x_L_ strikingly inhibits invasiveness of CRC cells. In this previous study, a knockdown of a single Bcl-2 protein (Mcl-1, Bcl-2 or Bcl-x_L_) was sufficient to block migration and invasion [7].

Based on these earlier reports, our study aimed at investigating the potential of Bcl-2 inhibiting small molecules as drug candidates for CRC treatment *in vitro*. First, we compared the efficacy of the Bcl-2 and Bcl-x_L_ inhibitor ABT-737 with the pan-Bcl-2-inhibitor Obatoclax. A direct comparison of the two inhibitors allows distinguishing effects of a broad pan-Bcl-2 inhibition by Obatoclax from a more specific way using ABT-737. Despite the fact that both inhibitors showed significant toxicity in clinical trials, these compounds can be used as tools for preclinical *in vitro*-testing of Bcl-2 inhibition [19]–[22]. ABT-737 has been shown to synergize with Oxaliplatin and Celecoxib in killing of CRC cells [23], [24]. We observed dose dependent apoptosis induction caused by ABT-737 in all cell lines investigated. It is well documented that a resistance towards ABT-737 can be driven by high levels of Mcl-1 via inhibition of proapoptotic NOXA [25]. Very recently, it has been demonstrated that cancer cells in hypoxic conditions are resensitized to ABT-737 by a loss of Mcl-1 [26]. Since effects of ABT-737 are clearly proapoptotic and determinants for sensitivity in CRC are well documented, we decided to further focus on antitumor effects of Obatoclax.

In sharp contrast to ABT-737, Obatoclax did not lead to significant cell death induction in CRC cells. In our study, Obatoclax doses applied were clinically relevant, as indicated in phase I trials, and did not reach the Compound IC_50_ reported for Obatoclax [19], [27] Interestingly, Obatoclax treatment resulted in stable or increasing expression levels of all antiapoptotic Bcl-2 proteins investigated. Another study showed downregulation of antiapoptotic Bcl-2 proteins under Obatoclax treatment in lymphoma cells [10]. Since this study demonstrates apoptosis of lymphoma cells caused by Obatoclax, decreased levels of antiapoptotic proteins might be secondary in the course of cell death rather than triggered by a direct binding effect of Obatoclax.

Knockdown or overexpression of Mcl-1, Bcl-2 or Bcl-x_L_ did not delay cell cycle progression and proliferation in CRC cells [7]. By contrast, low dose Obatoclax treatment caused delayed proliferation in our study. We discovered a G1-phase arrest accompanied by loss of Cyclin D1 expression and upregulation of p21 and p27. Cyclin D1 (CD1) is the most prominent G1-phase Cyclin and has been reported as an oncogenic driver in cancer cells. CD1 expression is associated with neoplastic transformation and increased malignancy in cancer [12]. The G1/S-phase transition is tightly regulated by CDKIs such as p21 and p27 via inactivation of G1 Cyclin-Cyclin dependent kinases (CDK) complexes [13], [28]. Taken together, our data reveal a novel cell cycle regulating property of Obatoclax via G1-phase arrest. This effect was not antagonized by overexpression of antiapoptotic Bcl-2 proteins. Furthermore, neither overexpression of antiapoptotic Bcl-2 proteins nor ABT-737 affected cell cycle progression. Delaying cell cycle and impairing uncontrolled growth of CRC cells is apparently a Bcl-2 protein independent effect of Obatoclax. Even if the exact underlying mechanisms and the relevant targets remain elusive, it might be possible that mTOR signaling plays a role in this context. A binding activity of Obatoclax to mTOR as well as a late stage autophagy inhibition have been recently reported and could play a causative role for the described antiproliferative effects [29], [30]. Further decent molecular analyses are warranted to dissect Obatoclax relevant impact on cell cycle regulation. For instance, CDKs may be investigated as potential targets of Obatoclax in future studies.

Conventional chemotherapeutics are most effective on replicating cancer cells. Even if we clearly demonstrate that Obatoclax reduces the proliferative properties of CRC cells, our data proves Obatoclax capacity to overcome cell death resistance towards Oxaliplatin in SW480 cells. The therapeutic potential of combining platin-based chemotherapy regimens with Obatoclax in order to overcome apoptosis resistance has already been reported and is further highlighted by our data [31], [32]. In esophageal carcinoma cells, Obatoclax had synergistic activities along with 5-FU [32]. In our study, synergistic effects of Obatoclax were restricted to Oxaliplatin pointing on a cancer cell type dependent effect. Strong effects of Obatoclax on autophagy have been demonstrated in several recent studies [32]–[34]. However, the relevance of Obatoclax-induced autophagy signaling remains elusive for CRC.

Migration and invasion are prerequisites of cancer cell spread, leading to local invasion and distant organ metastasis [35]–[37]. Our group recently demonstrated regulatory functions of antiapoptotic Bcl-2 proteins on migration and invasion of CRC cells independent of cell death and proliferation [7]. In the light of the fact that Obatoclax could not sufficiently induce death in CRC cells, we investigated migration and invasion of CRC cells under Obatoclax treatment. Strikingly, low dose Obatoclax blocked migration and invasion in all cell lines investigated. Long term 3D cell culture of CRC cells under Obatoclax treatment confirmed this blockade of migration despite a lack of cell death induction. Importantly, migration was blocked by Obatoclax in resistant cell lines such as Colo205 and CaCo2. Cadherins are important regulators of cell attachment and are involved in major signaling networks such as Wnt. It has been shown that a conditional intestinal specific knockout of E-Cadherin caused increased migration and proliferation in the intestine [38]. On the other hand, expression of E-Cadherin slowed migration and proliferation in the intestinal crypts [39]. In colorectal carcinogenesis, functional elimination of E-Cadherins represents a key step in the acquisition of invasiveness [40]. We, therefore, aimed to investigate changes in E-Cadherin expression. We demonstrate a profound restoration of E-Cadherin in CRC cells under treatment with Obatoclax. However, E-Cadherin upregulation may represent the molecular switch back to a less invasive phenotype of CRC cells caused by Obatoclax.

Importantly, and in contrast to the growth inhibitory function of Obatoclax, migration was completely restored in CRC cells overexpressing Mcl-1, Bcl-2 or Bcl-x_L_. In the context of invasiveness, antiapoptotic Bcl-2 proteins appear as the main affected targets. This is in line with our earlier report showing a regulatory function of antiapoptotic Bcl-2 proteins on CRC cell invasiveness without affecting proliferation or inducing cell death [7]. Other studies support the hypothesis that Bcl-2 proteins are relevant for metastasis [41]–[43]. Conversely, ABT-737 treatment is not sufficient to block migration and invasion of CRC cells. In our study, we show that Obatoclax is capable of inhibiting both, migration and invasion, even in very low doses. This effect of Obatoclax is novel and considerably, even in primarily resistant cells. Since ABT-737 does not inhibit migration, we propose an agent-specific and unique feature of Obatoclax within the group of compounds targeting Bcl-2 proteins.

## Conclusion

Based on the data of our study, we conclude that the Pan-Bcl-2 inhibitor Obatoclax counteracts various biological processes relevant for tumor progression. The efficacy of Obatoclax on CRC cells is broad and includes a cell death independent but Bcl-2 protein addicted inhibition of migration. The second major effect affects cell cycle progression and is independent of Bcl-2 protein targeting. Thus, pan-Bcl-2 inhibition, including the development of specific and less toxic inhibitors, is a promising approach for CRC treatment and should be further analysed, e.g. in combination with chemotherapy.

## Material and Methods

### Reagents and cell lines

CRC cell lines HT29, SW480, CaCo2 and Colo205 were purchased from ATCC. Cells were cultured in a humidified atmosphere (37°C, 5% CO_2_) in RPMI + Glutamax (Gibco, Karlsruhe, Germany) supplemented with 10% FCS (PAA Laboratories, Cölbe, Germany), 1% Pen/Strep (PAA Laboratories), 1% HEPES (Gibco) and 1% Nonessential amino acids (NEAA, Gibco). Obatoclax and ABT-737 were purchased from Selleckchem (Munich, Germany), Oxaliplatin from Sigma-Aldrich (Munich, Germany).

### Viability and cell growth test

Cells were seeded into 12 well plates and 24 h after seeding transfected or treated as indicated. Cell growth was determined using a colorimetric 3-(4, 5-Dimethylthiazol-2-yl)-2, 5-diphenyltetrazolium bromide (MTT) assay as described [7].

### Quantitative real-time polymerase chain reaction (q-RT PCR)

Isolation of total RNA and cDNA synthesis was performed as previously described [44]. Q-RT PCR was performed using primer assay kits (Qiagen, Hilden, Germany). Data acquisition and determination of gene expression was performed using the LightCycler software package (Roche, Mannheim, Germany). Each PCR reaction was run in duplicates. mRNA expression was normalized to the expression of the housekeeping gene GAPDH.

### Detection of apoptosis and cell cycle phase distribution

On day one after transfection, cells were treated as indicated for 48 h. Supernatant was transferred to FACS tubes and cells were then gently detached using Accutase. After centrifugation, cells were resuspended in a hypotonic buffer containing 0.1% (w/v) sodium citrate, 0.1% (v/v) Triton X-100 and 50 µg/ml Propidium iodide (Sigma-Aldrich). After 1 h incubation at 4°C, total DNA content of cells was measured according to the protocol of Nicoletti *et al.* using flow cytometry [45]. Cell cycle analysis was performed using FACS Diva 6 (Becton Dickinson) and FlowJo 7.6.5. (Tree Star Inc.). Cells representing the sub-G1 fraction were depicted as apoptotic.

### Cell lysis, SDS-Page, Western blotting and densitometry

Cells were seeded into 6 well plates, cultured for 24 h and treated as indicated. Cell lysis, SDS-Page and Western blotting were performed as described previously [46]. The following antibodies were used for immunodetection: anti-Mcl-1 (Santa Cruz biotechnology, Heidelberg, Germany), anti-Bcl-x_L_ (Cell Signaling, Boston MA USA), anti-Bcl-2 (Abcam, Cambridge, UK), anti-E-Cadherin, anti-p27, anti-p21, anti-cleaved PARP (all Cell Signaling) and anti-Tubulin (Sigma-Aldrich).

To quantify the protein bands we used the ImageJ software for densitometric analysis. The band density was measured relative to the untreated control and then adjusted to tubulin as loading control.

### Transfection

Plasmid transfection was performed using Lipofectamine LTX (Invitrogen) in OptiMEM for SW480 cells or peqFECT DNA (Peqlab, Erlangen, Germany) in complete RPMI for HT29 cells as described previously [7]. The following plasmids were used: human Mcl-1, Bcl-2 and Bcl-x_L_ were cloned in a pcDNA3 vector. pcDNA3-hBCL-2 was a kind gift of W. Roth (Institute of Pathology, Heidelberg). pcDNA3-hBCL-x_L_ was kindly provided by M. Li-Weber (German Cancer Research Center, Heidelberg, Germany). Corresponding empty vector were used as controls.

### Migration assay

2×10^6^ cells were seeded into 6 well plates and grown to a confluence of about 70–80%. The cell monolayer was scratched using a sterile pipette tip. Cells were then washed with medium and images were immediately captured using an inverted microscope (CKX41, Olympus Inc., Hamburg, Germany) equipped with a digital color camera (XC30, Olympus Inc.). The exact location of the image within the monolayer was marked to identify the same gap over the next 48 h. The gap closure was measured every 24 h as follows using CellSense imaging software (Olympus Inc.): Gap distances of the scratch between one side and the other were measured at certain intervals along the edge of the generated scratch (every 200 µm). The mean of the measured distances was then calculated and compared to the mean distance of the gap at the starting time point of the experiment [47].

### Invasion assay

BioCoat Matrigel invasion chambers (8 micron pore size, BD) were used to study invasion of SW480 cells. Matrigel invasion chambers were prepared and proceeded as described before [7]. Cells were harvested using Accutase (PAA), pooled and counted. 3×10^5^ cells were then resuspended in 500 µl FCS free RPMI containing the indicated concentration of Obatoclax mesylate and transferred into the upper part of the invasion chamber. After 72 h, nuclei of invaded cells were stained using Hoechst 33342 (Invitrogen) for 10 min. Five pictures of every insert were taken and the number of invaded cells was counted by the naked eye.

### 3D cell culture

We used Alvetex Scaffolds made of a cross-linked polystyrene scaffold as described before [7]. Medium was changed and freshly prepared drug added every 48 h. DMSO served as vehicle. After 7 days, scaffolds were further processed as described previously [7]. In addition, immunhistochemistry was performed using NovoLink Polymer detection System (Leica Microsystems, Wetzlar, Germany) according to the manufacturer's instructions after 4% PFA-fixation and heat induced epitope retrieval (HIER) in citrate buffer (pH 6). Antibodies against Ki67 (Abcam), and cleaved PARP (Cell Signaling) were used. Images were captured using an inverted microscope. Images were analyzed using CellSense and ImageJ software.

### Cell Counting

2×10^6^ cells were seeded into 6 well plates and transfected with specific expression plasmids for Mcl-1, Bcl-x_L_ or Bcl-2 after 24 h as described. Cells were harvested, resuspended and then counted using a Neubauer chamber 24, 48 and 72 h post transfection as described [7].

### Statistical analysis

Data obtained in invasion and 3D-scaffold experiments were analyzed using the Student's t-test (paired, two-sided) based on normal data distribution. For migration assays, the relationship between gap closure as response, and time and treatment as explanatory variables was investigated by an analysis of variance (ANOVA). SPSS 20 statistics (IBM, NY USA) software was used for all statistic analyses. A value of p<0.05 was considered as significant.

## Supporting Information

Figure S1 **Migration of HT29 cells treated with ABT-737.** (**A**) Gap closure of HT29 cells treated with 0.5 µM ABT-737 for 72 h. (**B**) Representative pictures of closing gaps corresponding to (**A**). (**C**) Gap closure of HT29 cells treated with 5 µM ABT-737 for 72 h. Values are expressed as mean ± SD. Assays are representative of at least three independent experiments.
(TIF)

Figure S2 **Long term Obatoclax treatment of Colo205 cells in 3D scaffolds.** (**A**) Colo205 cells were seeded into scaffolds and treated with Obatoclax for 7 days. Left panel: Ki67 staining of vehicle and Obatoclax treated Colo205 cells. Right panel: Cleaved PARP staining of vehicle and Obatoclax treated Colo205 cells. (**B**) Graphs for viability, total cell count and invasion depth for Colo205 cells treated with Obatoclax for 7 days in 3D scaffolds. Values are expressed as mean ± SD. Assays are representative of at least three independent experiments. Oba  =  Obatoclax.
(TIF)

Figure S3 **Apoptosis in Obatoclax and Oxaliplatin treated CRC cells.** (**A–B**) HT29 cells and SW480 cells were seeded onto 12 well plates and treated with vehicle, Oxaliplatin (10 µM), Obatoclax (0.25 µM) or Oxaliplatin (10 µM) and Obatoclax (0.25 µM). After 48 h, cells were harvested and subjected to flow cytometric analysis for apoptotis as described. (**C**) SW480 cells were seeded into scaffolds and treated with Obatoclax (0.25 µM) and Oxaliplatin (20 µM) for 7 days. Representative pictures (left) and corresponding analysis (right) for cleaved PARP staining of vehicle and Obatoclax or Oxaliplatin treated SW480 cells. Values are expressed as mean ± SD. Assays are representative of at least three independent experiments. Oba  =  Obatoclax, oxa  =  Oxaliplatin.
(TIF)

Figure S4 **Proliferation in CRC cells overexpressing antiapoptotic Bcl-2 proteins.** (**A**) HT29 cells overexpressing Bcl-2. 3-D scaffolds sectioned and stained for Ki67 after 4 days. Scale bar indicates magnification for both panels. (**B**) Corresponding total cell count (upper graph), Ki67 positivity (%, middle graph) and cl. PARP positivity (%, lower graph). (**C**) Western blot of SW480 cells after transfection with Mcl-1, Bcl-2 and Bcl-x_L_ expression plasmid. (**D**) Cell counting of SW480 after transfection with either vector or expression plasmids for Mcl-1, Bcl-2 or Bcl-x_L_. All assays are respresentative for at least three independent experiments. Bars represent mean ± SD. Vec  =  vector.
(TIF)
