# Supplementary figures and images for: Pan-Bcl-2 Inhibitor Obatoclax Delays Cell Cycle Progression and Blocks Migration of Colorectal Cancer Cells
Source: PLoS One. 2014 Sep 5;9(9):e106571. doi: 10.1371/journal.pone.0106571 (PMC4156353; doi:10.1371/journal.pone.0106571)

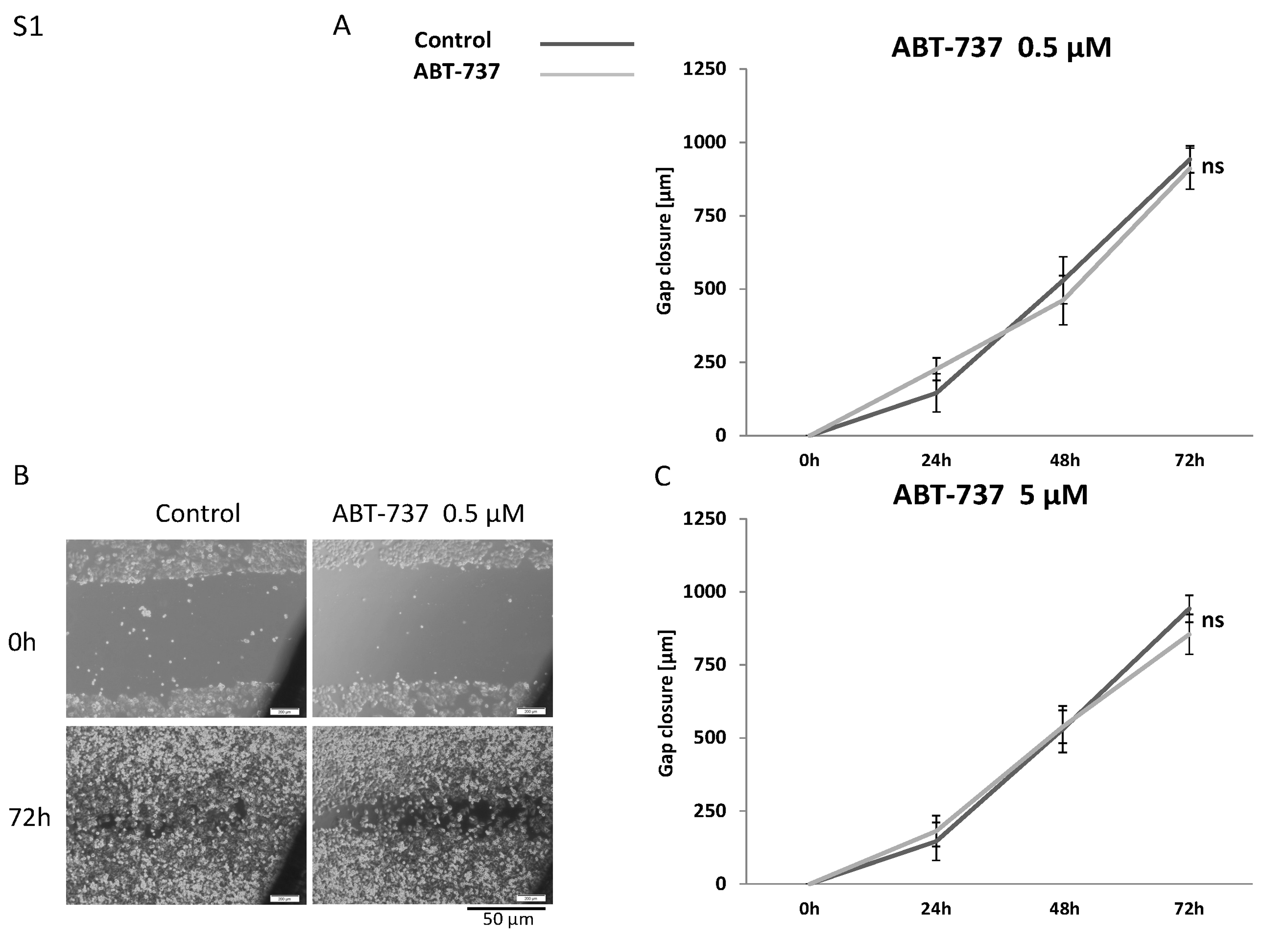

Supplement: Figure S1 — Migration of HT29 cells treated with ABT-737. (A) Gap closure of HT29 cells treated with 0.5 µM ABT-737 for 72 h. (B) Representative pictures of closing gaps corresponding to (A). (C) Gap closure of HT29 cells treated with 5 µM ABT-737 for 72 h. Values are expressed as mean ± SD. Assays are representative of at least three independent experiments. (TIF) [file pone.0106571.s001.tif]

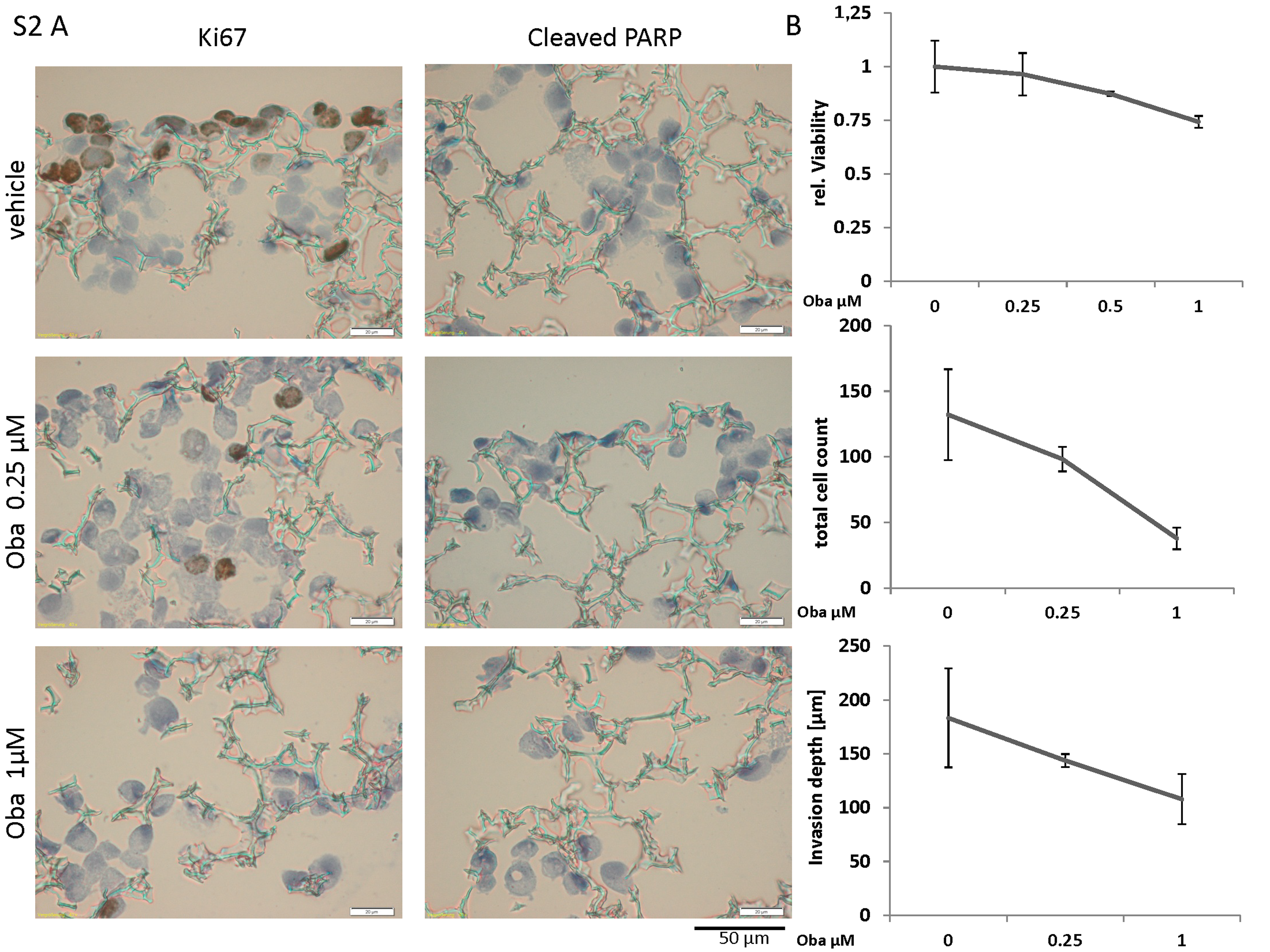

Supplement: Figure S2 — Long term Obatoclax treatment of Colo205 cells in 3D scaffolds. (A) Colo205 cells were seeded into scaffolds and treated with Obatoclax for 7 days. Left panel: Ki67 staining of vehicle and Obatoclax treated Colo205 cells. Right panel: Cleaved PARP staining of vehicle and Obatoclax treated Colo205 cells. (B) Graphs for viability, total cell count and invasion depth for Colo205 cells treated with Obatoclax for 7 days in 3D scaffolds. Values are expressed as mean ± SD. Assays are representative of at least three independent experiments. Oba = Obatoclax. (TIF) [file pone.0106571.s002.tif]

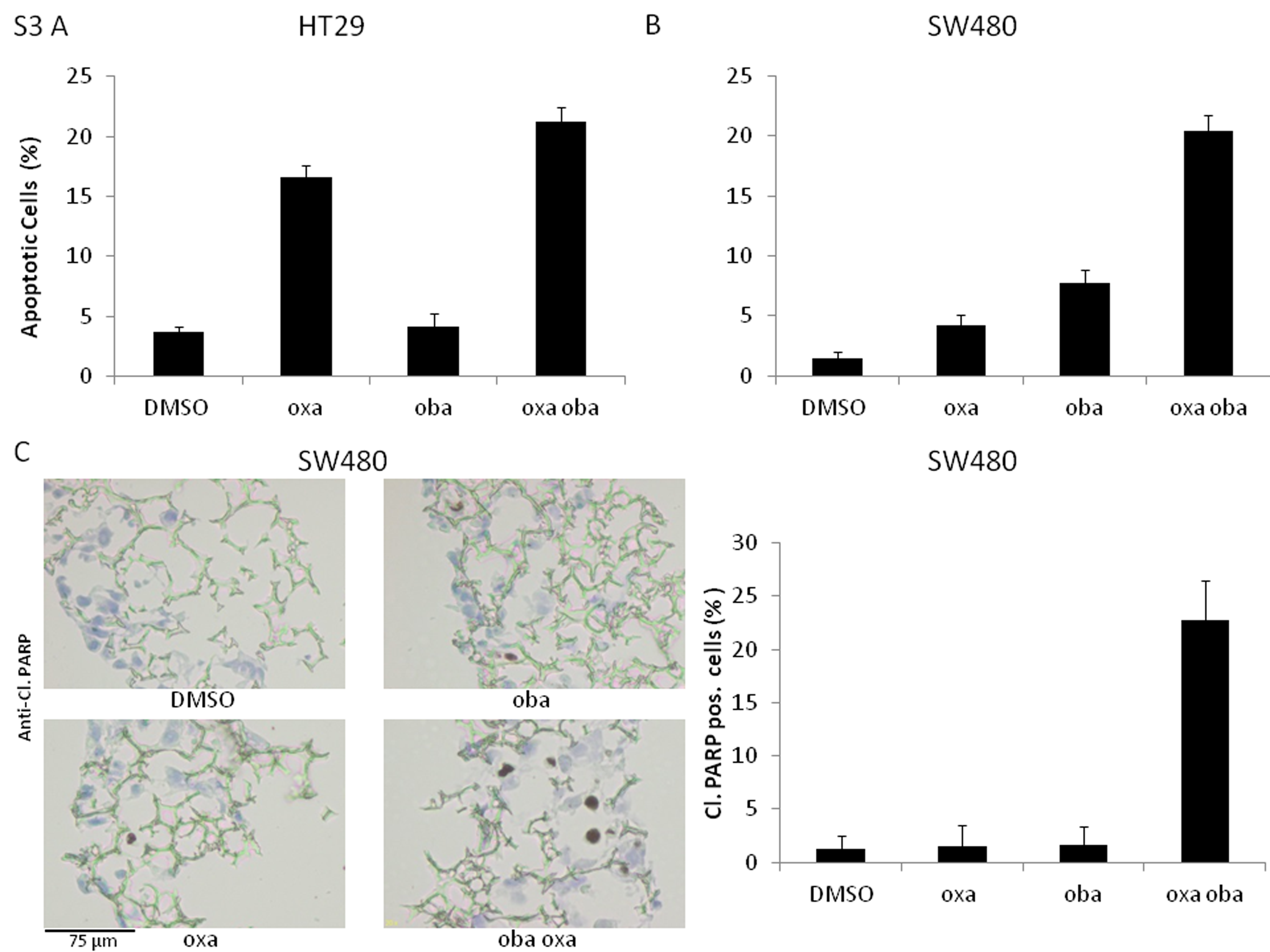

Supplement: Figure S3 — Apoptosis in Obatoclax and Oxaliplatin treated CRC cells. (A–B) HT29 cells and SW480 cells were seeded onto 12 well plates and treated with vehicle, Oxaliplatin (10 µM), Obatoclax (0.25 µM) or Oxaliplatin (10 µM) and Obatoclax (0.25 µM). After 48 h, cells were harvested and subjected to flow cytometric analysis for apoptotis as described. (C) SW480 cells were seeded into scaffolds and treated with Obatoclax (0.25 µM) and Oxaliplatin (20 µM) for 7 days. Representative pictures (left) and corresponding analysis (right) for cleaved PARP staining of vehicle and Obatoclax or Oxaliplatin treated SW480 cells. Values are expressed as mean ± SD. Assays are representative of at least three independent experiments. Oba = Obatoclax, oxa = Oxaliplatin. (TIF) [file pone.0106571.s003.tif]

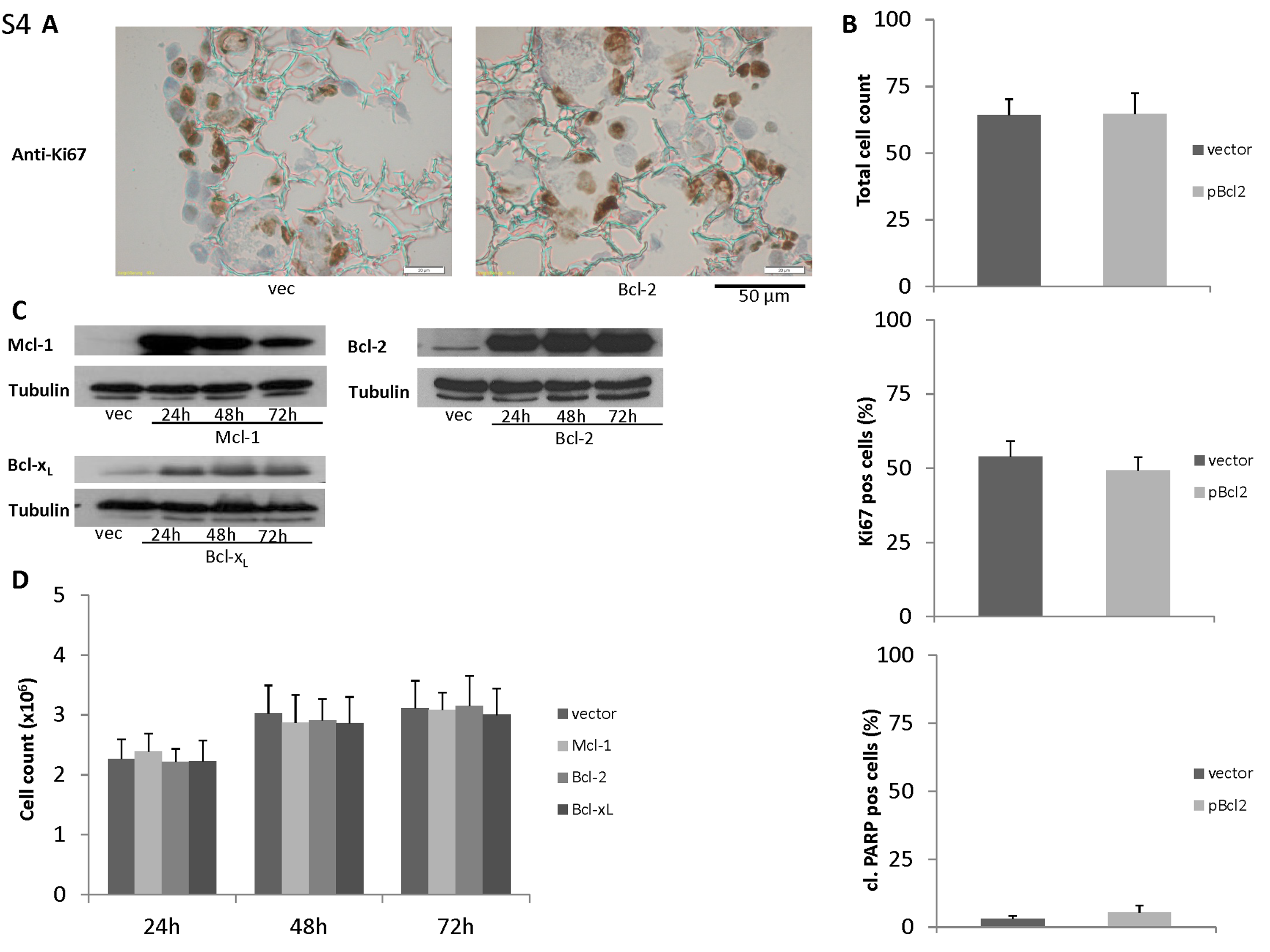

Supplement: Figure S4 — Proliferation in CRC cells overexpressing antiapoptotic Bcl-2 proteins. (A) HT29 cells overexpressing Bcl-2. 3-D scaffolds sectioned and stained for Ki67 after 4 days. Scale bar indicates magnification for both panels. (B) Corresponding total cell count (upper graph), Ki67 positivity (%, middle graph) and cl. PARP positivity (%, lower graph). (C) Western blot of SW480 cells after transfection with Mcl-1, Bcl-2 and Bcl-xL expression plasmid. (D) Cell counting of SW480 after transfection with either vector or expression plasmids for Mcl-1, Bcl-2 or Bcl-xL. All assays are respresentative for at least three independent experiments. Bars represent mean ± SD. Vec = vector. (TIF) [file pone.0106571.s004.tif]
